# Roles of Peroxisomes in the Rice Blast Fungus

**DOI:** 10.1155/2016/9343417

**Published:** 2016-08-16

**Authors:** Xiao-Lin Chen, Zhao Wang, Caiyun Liu

**Affiliations:** State Key Laboratory of Agricultural Microbiology, The Provincial Key Lab of Plant Pathology of Hubei Province, College of Plant Science and Technology, Huazhong Agricultural University, Wuhan 430070, China

## Abstract

The rice blast fungus,* Magnaporthe oryzae*, is a model plant pathogenic fungus and is a severe threat to global rice production. Over the past two decades, it has been found that the peroxisomes play indispensable roles during* M. oryzae* infection. Given the importance of the peroxisomes for virulence, we review recent advances of the peroxisomes roles during* M. oryzae* infection processes. We firstly introduce the molecular mechanisms and life cycles of the peroxisomes. And then, metabolic functions related to the peroxisomes are also discussed. Finally, we provide an overview of the relationship between peroxisomes and pathogenicity.

## 1. Introduction

Peroxisomes are single membrane-bound microbodies which existed in all eukaryotic cells. In different organisms and environmental conditions, their abundance can be changed rapidly, and functions could be different. The abundance of peroxisomes is coordinated by several cellular processes, including peroxisome biogenesis, peroxisome proliferation, and peroxisome degradation [1]. In budding yeast, the genes involved in those cellular processes include a set of* PEX* genes, which encode peroxins [2]. Up to now, over 30* PEX* genes have been found in different organisms [2].

There are several important metabolic processes that take place in peroxisomes, which involve fatty acids *β*-oxidation, glyoxylate cycle, hydrogen peroxide detoxification and secondary metabolite biosynthesis, and so forth [1]. The roles of peroxisomes have been extensively uncovered in yeast, filamentous fungi, plant, and human [3]. Some peroxisome functions are species specific, such as the methanol assimilation in yeast [4], the glyoxylate cycle in plant seeds [5], and the plasmalogens biosynthesis in mammal [6]. In filamentous fungi, they can develop a special compartment, the peroxisome-derived woronin body, to seal the septal pore when suffered cellular wounding [7].

The molecular mechanisms of peroxisome life cycle have been extensively studied in yeast [2]. Peroxisome studies in the filamentous fungi are also increased rapidly, especially in the model filamentous fungi* Aspergillus nidulans* [8, 9] and* Neurospora crassa* [10–12], the plant pathogens* Colletotrichum orbiculare* [13–15] and* Magnaporthe oryzae* [16–20], and the human pathogens* Candida albicans* [21, 22],* Aspergillus fumigatus* [23, 24], and* Cryptococcus neoformans* [25, 26]. An important aspect is that the peroxisomes are found to play key roles in fungal pathogenicity towards their host, including plants and human. In all these pathogenic fungi, the roles of peroxisomes in* M. oryzae* have received extensive concern.

Over the past two decades, many components involving roles of peroxisomes have been identified in* M. oryzae* (Table 1). This review focuses on recent advances in our understanding of peroxisomes in* M. oryzae*, including a description of the peroxisome life cycle during fungal infection, and an overview of their remarkable functions relevant to pathogenesis.

## 2. Life Cycle of the Peroxisomes

The peroxisome life cycle mainly includes peroxisome biogenesis, peroxisome proliferation, and peroxisome degradation [27]. The peroxisomes are thought to be born originally from the endoplasmic reticulum (ER) [27]. Basically, the biogenesis process contains peroxisomal membrane proteins (PMPs) acquisition and peroxisomal matrix proteins import. Peroxisome proliferation can be achieved by either* de novo* formation from ER or/and peroxisome fission. When the peroxisomes have finished their mission, they can be degraded by pexophagy, an autophagic process [27].

In* M. oryzae*, there are several* PEX* genes involving peroxisome life cycle that have been characterized, including* MoPEX5*,* MoPEX6*,* MoPEX7*,* MoPEX14*,* MoPEX19,* and* MoPEX11* family genes.* MoPEX5* and* MoPEX7* are involved in matrix proteins import [17, 18],* MoPEX6* participates in receptors import for recycling [28],* MoPex14* functions as a matrix docking protein [19],* MoPEX19* functions as chaperone and receptor for importing of both matrix proteins and PMPs [16], and* MoPEX11* family genes are involved in peroxisomal fission processes [20].

### 2.1. Peroxisome Biogenesis

In yeast, during peroxisome biogenesis, peroxisome membrane proteins (PMPs) should firstly be inserted into membranes, which are mediated by* PEX3*,* PEX16,* and* PEX19* [29–31]. Then the peroxisomal matrix proteins, which are synthesized in the cytoplasm, are translocated into the peroxisomes by peroxisome membrane docking complex [32]. Most of the peroxisomal matrix proteins contain either PTS1 (type I peroxisomal targeting signal) at the C-terminus or PTS2 (type II peroxisomal targeting signal) at the N-terminal and can be recognized by shuttle receptors Pex5 or Pex7-mediated complex, respectively. The cargos on the PTS1 and PTS2 receptors are accepted by the Pex13/Pex14/Pex17 docking complex, and then the receptors are recycled by the ubiquitin system. The ubiquitinated receptors can be extracted into cytoplasm by AAA+ ATPases Pex1 and Pex6 [27].

In* M. oryzae*, MoPex19 is the ortholog of yeast PMPs receptor Pex19. Pex19 protein is located in the cytoplasm and newly formed peroxisomes, which is consistent with its PMPs receptor function that shuttles the PMPs between the cytosol and peroxisomal membrane [30]. Deletion of* MoPEX19* will lead to PMPs mislocalization. PMP47 is a representative PMP which is normally distributed in the peroxisomes in the wild type strain, while in the* MoPEX19* deletion mutants, its localization pattern is changed, which is distributed in the cytoplasm [16]. This demonstrated that the PMP47 cannot be imported into the peroxisomes. Moreover, in the Δ*mopex19* mutants, peroxisomal structures and peroxisome-derived woronin bodies are both absent [16], indicating that the MoPex19 is essential for biogenesis of peroxisomes and woronin bodies.

*M. oryzae* MoPex5 and MoPex7 are also proved to function as receptors of peroxisomal matrix proteins, which are involved in importing of the matrix proteins into peroxisomes [17, 18]. Disruption of* MoPEX5* and* MoPEX7* will block the PTS1 and PTS2 peroxisomal import pathways, respectively. In the wild type strain, RFP-PTS1 and GFP-PTS2 are normally distributed in the punctuate peroxisomes, while in the Δ*mopex5* mutants, RFP-PTS1 is dispersed in cytoplasm but GFP-PTS2 is still located in peroxisomes. In contrast, in the Δ*mopex7* mutants, RFP-PTS1 is still located in peroxisomes but GFP-PTS2 is dispersed in cytoplasm. These results demonstrated the MoPex5-mediated PTS1 peroxisomal import pathway and MoPex7-mediated PTS2 peroxisomal import pathway separately function in the rice blast fungus [17, 18]. The thiolase MoThl1 is a candidate PTS2 protein; it is failed to be located at the peroxisomes in the Δ*mopex7* mutants [17], which further supports the role of MoPex7 in the PTS2 peroxisomal protein import pathway.

The function of Pex6 ortholog in* M. oryzae*, MoPex6, was also evaluated [28]. In* MoPEX6* disruption mutants, the GFP-SRL protein is diffused in the cytoplasm, failed to be localized in the punctuate peroxisomes in mycelia, conidia, germ tubes, and appressoria [28], indicating the PTS peroxisomal import pathway is blocked. This result is consistent with the cellular function of Pex6, which is involved in recycling of matrix protein receptors (Pex5 and Pex7) during peroxisome biogenesis.

In the* MoPEX14* disruption mutants, the GFP-SRL protein is also mislocalized to the cytoplasm, while when the* PEX14*_*61-361*_ or* PEX14*_*1-258*_ is expressed in the Δ*mopex14* mutants, the punctate localization of GFP-SRL can be restored [19]. These data confirmed the functions of* MoPex14*, which act as a matrix docking protein to facilitate peroxisomal protein import and peroxisome biogenesis.

### 2.2. Peroxisome Proliferation

Peroxisomes can proliferate rapidly according to suitable environment stimulation. The proliferation processes can be achieved by* de novo* biogenesis from the ER, or by fission from the preexisting peroxisomes. In yeast, the peroxisome fission processes mainly consist of several steps. At the beginning, the mature peroxisomes are elongated by the functions of the peroxisomal membrane protein Pex11. Then the matrix proteins can be imported into the elongated peroxisomes, and the fission machinery can also be imported into appropriate place for fission. The dynamin-like protein Dnm1 is located at the constriction sites and leads to membrane fission processes by GTP hydrolysis. At last, the daughter peroxisomes can be produced from the fission processes, which is achieved by cooperation of several proteins, including Fis1, Dnm1, and the adaptors Mdv1 or Caf4 [27].

The peroxisome fission process in* M. oryzae* is identified recently, by several independent studies. There are three members of* PEX11 *family genes in* M. oryzae* genome, named* MoPEX11A*,* MoPEX11B,* and* MoPEX11C*, respectively [20]. However, it seems that only* MoPEX11A* plays vital role in peroxisome fission. The* MoPEX11A* deletion mutant exhibits decreased but enlarged peroxisomes compared to the wild type, which demonstrated the* MoPEX11A* is important for peroxisome elongation and proliferation. In contrast, the* MoPEX11B* and* MoPEX11C* deletion mutants are normal in both number and size of the peroxisomes [20], indicating they could not be key regulators during peroxisomal proliferation.

There is only one counterpart of Mdv1/Caf4 protein in* M. oryzae*, named* PEF1* [peroxisome fission protein 1] [33]. This gene may play dual roles of Mdv1 and Caf4, because deletion of* PEF1* will lead to evident peroxisomal fission defect during the fission inducing conditions. The Δ*pef1* mutant forms string-linked peroxisomes, in contrast to the punctuate structures in normal cells [33]. Similar situation can be found in the Δ*mdv1*Δ*caf4* double mutant or the Δ*fis1* and Δ*dnm1* mutants in yeast [34]. The phenotypic defect indicates that the daughter peroxisomes cannot be cut from the elongated peroxisomes in Δ*pef1*. Pef1 can bind to Fis1 with its N-terminal extension (NTE) region and to Dnm1 with its C-terminal WD40 repeat region. With the help of adaptors Mdv1 or Caf4, the outer membrane protein Fis1 can recruit Dnm1 to peroxisomes for fission [35]. Pef1 can be well colocalized with MoFis1, which is recently reported to play important roles in mitochondria fission in* M. oryzae* [36]. This is intelligible, because in* S. cerevisiae*, the peroxisome fission machinery can be also used to facilitate mitochondria fission process.

### 2.3. Peroxisome Degradation

Peroxisome abundance can be rapidly cleared by a selective autophagic process, which is known as the pexophagy. In yeast, there are two different peroxisome degradation modes, macropexophagy and micropexophagy [37]. The macropexophagy sequesters peroxisomes to form a pexophagosome, which leads the peroxisomes to vacuole for degradation. The micropexophagy encloses peroxisomes by vacuolar membrane protrusions and the micropexophagy specific membrane apparatus (MIPA) to vacuole for degradation [37]. There are several* ATG* and* PEX* genes reported to be involved in perophagy [37].

In* Pichia pastoris*,* ATG30* is required for both of macropexophagy and micropexophagy [38], but no* ATG30* homolog gene can be found in* M. oryzae*. In* P. pastoris* and* C. orbiculare*,* ATG26* plays key roles in pexophagy, and the* CoATG26* is essential for infection process [13, 39]. However, In* M. oryzae*,* MoATG26* is not involved in pexophagy and is dispensable for virulence [19].

In* S. cerevisiae*, the pexophagy process can be mediated by Atg20/Snx42 [40]. By the assistance of sorting nexins Snx41 and Atg24/Snx4, Atg20/Snx42 can be also involved in endosomal retrieval trafficking [40]. However, only one protein [MoSnx41] with high similarity to Snx41 and Snx42/Atg20 can be found in* M. oryzae*. Studies have found that the* MoSNX41* plays key roles in conidiation and pathogenesis. Deletion of* MoSNX41 *leads to pexophagy deficiency, demonstrating this gene is involved in pexophagy in* M. oryzae*. The yeast ScSnx42 can restore the pexophagy deficiency of the Δ*mosnx41* mutant but failed to recover the defects of conidiation and pathogenesis, indicating the pexophagy process is dispensable for development and pathogenicity in* M. oryzae*. The function of MoSnx41 in conidiation and pathogenesis is mediated by Snx41-dependent retrieval trafficking, but not pexophagy [19]. As a peroxisomal membrane protein,* PEX14* is also involved in pexophagy of* Hansenula polymorpha* [41]. In* M. oryzae*, the MoPex14 has also been proved to be essential for pexophagy, but dispensable for pathogenicity.

### 2.4. Peroxisome Differentiation

Filamentous fungi can form a special structure, called woronin body (WB), which is differentiated from the peroxisomes [42]. It is accepted that the peroxisome-related woronin bodies are used to seal the septal pore when suffering cellular wounding [43]. The formation mechanism of woronin bodies has been well studied in* Neurospora crassa* [44]. Hexagonal peroxisome Hex1 is the major structural protein in woronin body, which can be imported into the peroxisome matrix and assembled to form a woronin body core structure. This core structure can recruit WB sorting complex protein [WSC] into the peroxisome membrane. Then the nascent WB can be budded from the mother peroxisomes.

In* M. oryzae*, the* HEX1* homolog gene has been cloned and the roles of the woronin bodies have also been uncovered by analyses of the* MoHEX1* functions. The woronin body is proved to be required for development, appressoria formation, and infection hyphae growth and therefore is essential for pathogenicity in* M. oryzae* [45]. Ultrastructural analyses proved that the woronin bodies are located adjacent to septa of mycelia, germ tubes, and infection hyphae, but they are less observed in conidia. The* HEX1* deletion mutant is lack of woronin bodies, and when the cells are damaged, the cellular materials will bleed out through the septal pores. The* M. oryzae* Hex1 protein contains the PTS1 peroxisome targeting signal, which can help it locate into the peroxisomes. For the woronin bodies being formed from the peroxisomes, defects in peroxisome formation cycles could lead to defect in woronin body formation. Consistent to this hypothesis, nearly all disruption of the genes involving in peroxisome life cycle can result in woronin body deficiency [45].

### 2.5. Regulation of Peroxisome Dynamics

The peroxisomes in the cell are dynamics according to environmental conditions and development stages. Occupancy of the peroxisomes in a cell is determined by several processes, including peroxisome biogenesis, peroxisome proliferation [*de novo* formation and fission], and peroxisome degradation (pexophagy).* De novo* biogenesis and fission process are both used to increase peroxisomes numbers. However, how to coordinate* de novo* formation and peroxisomal fission remains unclear. It is possible that both of the ER formation and fission of the preexisting peroxisomes might exist in normal conditions. It is supposed that, upon the peroxisome fission inducing conditions, such as in fatty acids condition, the peroxisomes are needed to be largely produced in a short time and the* de novo* formation process may be not enough or effective. In contrast, the peroxisome fission cycles would fulfill peroxisome demands in a limited time. During infection of* M. oryzae*, the lipid stores should be utilized less than 12 h. The rapid lipolysis of lipid bodies produced mass fatty acids, which in turn induces peroxisome fission process (*de novo* biogenesis efficiency could also be elevated), and massive peroxisomes are produced in short time, thus promoting fatty acids utilization and facilitating infection. When fatty acids are utilized, the peroxisomes should be decreased in a short time, and then they can be degraded by pexophagy. In* C. orbiculare*, the CoAtg26-dependent pexophagy is used to recycle cellular amino acids of the appressoria for infection [13]. How the peroxisome fission and pexophagy are activated and suspended remains obscure.

The Snf1/AMPK pathway plays central role in response to nutrient stress in* M. oryzae*. A recent study demonstrated that the MoSnf1 pathway can regulate peroxisomal maintenance and lipid metabolism by responding to nutrient-free environment [46]. In Δ*mosnf1* mutant, the peroxisomes are significantly decreased during appressoria formation. Accordingly, the Δ*mosnf1* mutant is also defect in lipid droplet mobilization, fails to generate enormous turgor, and loses its virulence [46]. Other regulatory mechanisms should also be uncovered in the future.

## 3. Metabolic Functions of Peroxisomes

Multiple metabolic processes occur in the peroxisomes, which makes the peroxisomes play crucial role in fungal development and pathogenesis. Peroxisomal *β*-oxidation of fatty acids ubiquitously existed for all eukaryotes. The reactive oxygen species [ROS] homeostasis can be also mediated by peroxisomes. Peroxisomes can be involved in many other metabolic pathways, including glyoxylate cycle in plants and fungi [52], penicillin biosynthesis in* Penicillium chrysogenum* [53], and melanin biogenesis in filamentous fungi [54].

### 3.1. Fatty Acid *β*-Oxidation

The *β*-oxidation metabolism is mainly used to degrade fatty acids for nutrient and energy utilization [55]. This metabolic process involves four major enzymes, acyl-CoA oxidase, 2-enoyl-CoA hydratase, 3-hydroxyacyl-CoA dehydrogenase, and 3-ketoacyl-CoA thiolase. Through a four-step pathway mediated by these enzymes, the acetyl-CoA is produced [55]. The acetyl-CoA can be fed to the glyoxylate cycle and gluconeogenesis to produce nutrient or metabolites.

In* M. oryzae*, the Mfp1/Fox2 protein has been shown to play an important role in fatty acid metabolism and pathogenesis [47]. The Δ*mfp1* mutants cannot grow on olive oil or oleic acid as sole carbon sources, indicating its roles in fatty acids metabolism. The expression level of* MFP1* is significantly induced in the olive oil condition. The Mfp1GFP fusion protein is well colocalized with FoxA-RFP in the peroxisome-like punctuate structures [47]. Because the* A. nidulans* FoxA has been proved to be a *β*-oxidation enzyme and can be located in the peroxisomes, the* M. oryzae* Mfp1 should also be located in the peroxisomes and is required for fatty acid *β*-oxidation.

In the past, *β*-oxidation is thought to occur exclusively in peroxisomes in the filamentous fungi, while it can occur in both peroxisomes and mitochondria in mammal. However, recent studies demonstrated that the mitochondrial *β*-oxidation is also functional and important for the infection of the fungal pathogens [56]. In* M. oryzae*, Enoyl-CoA hydratase Ech1 is an important mitochondrial *β*-oxidation enzyme, which is important for conidial germination, appressoria penetration, and host colonization. The Δ*ech1* mutant cannot utilize C14 fatty acid and also cannot well utilize C16 and C18 fatty acids. Consequently, Δ*ech1* is reduced in melanization and sensitive to oxidative stress. Generally, the short- and medium-chain fatty acids (less than 20C) can be oxidized in mitochondria, while long-chain fatty acids (over 20C) should be degraded in peroxisomes to shorter chain fatty acids for full oxidation in mitochondria [56]. Thus, the mitochondrial *β*-oxidation and peroxisomal *β*-oxidation can collaborate with each other for different length fatty acid oxidation in* M. oryzae*.

Acetyl CoA is the main product of fatty acid *β*-oxidation in the appressorium of* M. oryzae*, which must be transported into different cellular compartments for consumption. The acetyl CoA transportation is catalysed by carnitine acetyl transferases (CATs). In budding yeast* S. cerevisiae*, Cat2 is involved in transferring acetyl CoA to the cytoplasm, while Cat1 is used to transfer acetyl CoA into mitochondria for utilization by the tricarboxylic acid cycle [57]. Two* CAT* genes,* PTH2* and* CRAT2,* have been studied in* M. oryzae* [48]. The results demonstrated that* PTH2* plays major role in acetyl CoA transfer. Pth2-GFP protein is colocalized with the peroxisome marker protein and is abundant in appressoria. The Δ*pth2* is reduced in melanin deposition, defect in host penetration, and essential for pathogenesis [48]. Further analysis found that the Δ*pth2* mutant cannot utilize some lipid substrates. In contrast, the Δ*crat2* displays no evident defect in those mentioned phenotypes [48]. The carnitine-acylcarnitine carrier protein Crc1 functions in transferring the acetyl CoA cross the mitochondrial membrane. The* M. oryzae MoCRC1* deletion mutant is severely reduced in appressorial penetration and invasive growth.* MoCRC1 *is also needed for utilization of olive oil [49].

### 3.2. Glyoxylate Cycle

The glyoxylate cycle is a metabolic pathway which can be normally found in plants and fungi [52]. This pathway can assimilate acetyl CoA for gluconeogenesis and eventually generate glucose. The glyoxylate cycle is mainly induced when the fatty acids and acetate should be used [52]. The fatty acid *β*-oxidation pathway produces massive acetyl CoA, which will be processed by glyoxylate cycle. In this pathway, the acetyl CoA can be converted to glyoxylate by isocitrate lyase, and then the glyoxylate can be further converted to malate by malate synthase. The malate can be further metabolized to hexoses by gluconeogenesis.

The isocitrate lyase (Icl1) and malate synthase (Mls1) are two of principal enzymes involving glyoxylate cycle [52]. In* M. oryzae*,* ICL1 *is highly expressed during appressoria formation and penetration stages, indicating that the glyoxylate cycle should be induced in these stages [50]. Defect in peroxisome biogenesis will lead to loss functions of glyoxylate cycle. For example, disruption of* MoPEX19* will result in failure in acetate utilization [16].

### 3.3. Redox Homeostasis

Oxidative reactions are theme of the peroxisome metabolism, which generates massive reactive oxidative species (ROS), especially the hydrogen peroxide (H_2_O_2_) [58]. In order to eliminate harmful ROS, ROS scavenging becomes an important peroxisomal metabolism. To detoxification, the hydrogen peroxide can be scavenged by catalases and peroxidases, which are abundant in peroxisomes. A number of catalases and peroxidases are predicted in genome of* M. oryzae*, and some of which (such as* CATB* and* CPXB*) have been reported to function in host ROS detoxification [59, 60]. However, no catalase or peroxidase has been revealed to take part in intracellular peroxisome ROS detoxification. Glutathione S-transferases (GSTs) and peroxiredoxins (PRXs) are other antioxidant enzymes existing in peroxisomes [61]. There is also no GST or PRX protein reported to play roles in peroxisome ROS detoxification. In* Alternaria brassicicola* and* A. fumigatus*, a transmembrane protein TmpL has been identified and proved to be important for reduction of intracellular ROS and detoxification of external ROS and thus is important for fungal infection. TmpL can be located in the woronin body, and its expression level is evidently elevated in conidiation and initial invasive growth stages [62].

During peroxisome *β*-oxidation process, acetyl-CoA formation will accompany mass formation of FADH2 and NADH. To keep peroxisome redox homeostasis, the FADH2 and NADH should be eliminated. For the NADH, it can be reoxidated to NAD^+^. This reaction can be catalyzed by the peroxisomal lactate dehydrogenase, which can mediate production of lactate from pyruvate. In* M. oryzae*, the pyruvate is generated by the alanine: glyoxylate aminotransferase 1 (AGT1), which transfers the alanine amino group to glyoxylate and results in formation of the pyruvate. AGT1GFP is colocalized with RFP-MTS1 fusion protein, demonstrating that AGT1 is located in the peroxisomes [51]. The Δ*agt1* mutant cannot form appressoria on artificial inductive surface. When the NAD^+^ and pyruvate were added during conidia germination on artificial inductive surface, the appressorium formation can be restored [51]. Thus, the AGT-mediated pyruvate generation can function as one of factors to maintain redox homeostasis during appressoria formation.

### 3.4. Melanin Biosynthesis

In filamentous fungi, peroxisomes not only are crucial for the primary metabolism, but also play important roles in the biosynthesis of secondary metabolites. Many plant pathogenic fungi can produce melanin to protect the conidia to survive in different environment and to facilitate host penetration during infection. The dihydroxy naphthalene (DHN) melanin is well studied and found to be essential for appressorial mediated penetration in* M. oryzae*. The fungal appressorium contains a distinct melanin layer located between the cell wall and the membrane, which can be used to generate turgor for penetration. The DHN melanin is synthesized by the polyketide pathway, through which the peroxisomes-derived acetyl-CoA can be used to produce the 1,3,6,8-tetrahydroxynaphthalene (1,3,6,8-THN). The 1,3,6,8-THN is then used to synthesize the DHN melanin, catalyzed by a series of enzymes, including Alb1, Rsy1, and Buf1 [63].

Because the acetyl-CoA is mainly produced in peroxisomes, defects in peroxisome formation would lead to block in melanin synthesis. Consistent with this prediction, all disruptions of the peroxisome biogenesis-related genes can result in melanin deficiency.

### 3.5. Cell Wall Biosynthesis

The fungal cell wall mainly consists of chitin, *β*-1,3-glucan, *β*-1,6-glucan, and mannoproteins [64]. This rigid structure can protect fungal from extracellular stresses and is flexible for adaptation to development and environment [64]. It is believed that fungal cell wall chitin and glucan are derived from acetyl-CoA, and the defects in peroxisomes will lead to deficiency in cell wall integrity. Consistent with this hypothesis, the* M. oryzae MoPEX5*,* MoPEX6, *and* MoPEX19* deletion mutants are all sensitive to the cell wall-perturbing agents, such as Congo Red and Calcofluor White [16, 18, 28].

## 4. Peroxisome and Pathogenicity

Functions of peroxisomes have been studied in kinds of organisms, including the filamentous fungi. In fungal pathogens, an intriguing feature is their roles in pathogenicity. It is reported that peroxisomes are required for virulence of almost all fungal pathogens, such as the plant pathogens* C. orbiculare* and* M. oryzae*, the insect pathogen* M. robertsii*, and the human pathogens* C. albicans*,* A. fumigatus* and* C. neoformans*.

**Figure 1:**
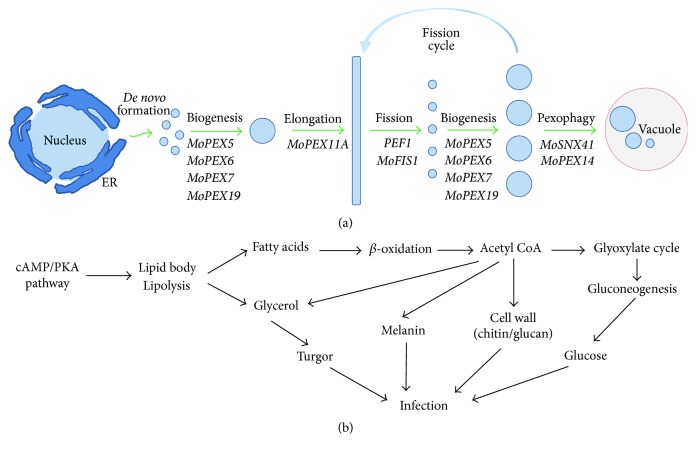
Life cycle and functions of the peroxisomes in* M. oryzae*. (a) Model of life cycle of the peroxisomes. The peroxisomes are synthesized from the ER and then mature through peroxisome biogenesis process. During the fission inducing condition, the matured peroxisomes can be elongated, and then the daughter peroxisomes can be produced by the fission process, the newly formed peroxisomes will mature through the biogenesis process, and the matured peroxisomes can be elongated again for another fission cycle. When the fission inducing condition is removed, the redundant peroxisomes can be eliminated through pexophagy process. (b) Function of the peroxisomes during fungal infection. When the conidia attach the host surface, the cAMP/PKA signaling pathway is activated, leading to mobilization of the lipid stores by lipolysis, and produces the fatty acids and glycerol. The glycerol will be used to generate turgor. The fatty acids will be taken by the peroxisomes for *β*-oxidation, which can produce mass of acetyl CoA. Acetyl CoA can be used by the glyoxylate cycle and gluconeogenesis to produce glucose for infection or be used to synthesize melanin and cell wall components. Together with the glycerol generated turgor, these products can help the fungus to penetrate the cuticle and colonize in the host cells.

During infection,* M. oryzae* can form a specialized structure known as appressorium. Along with the formation and maturation of appressoria, the lipid stores are mobilized and utilized. The lipid stores are firstly coupled to lipolysis, resulting in triglycerides and glycerol; the latter can accumulate enormous turgor pressure. Meanwhile, the triglycerides can be adopted by the peroxisomes for subsequent fatty acids *β*-oxidation to produce acetyl CoA and ATP. The acetyl CoA can be utilized by the glyoxylate cycle and gluconeogenesis pathway for glucan and chitin biosynthesis; they can also be utilized for melanin biosynthesis. Therefore, the peroxisomes mediated cellular processes and metabolisms can provide key factors, such as melanin and cell wall integrity, and play key roles during* M. oryzae* infection (Figure 1). Detailed peroxisome-related roles during* M. oryzae* infection will be described below.

### 4.1. Peroxisome Biogenesis and Pathogenesis

Defects in peroxisomal biogenesis will lead to severe disorder of peroxisomal metabolisms, including fatty acids *β*-oxidation, glyoxylate cycle, melanin, and cell wall biosynthesis. Consequently, the fungal development and pathogenicity will be severely affected. During germination and appressorial development, the expression of* MoPEX19* was evidently elevated. Deletion of this gene will lead to deficiency in glyoxylate cycle and severe defects in development and complete loss of pathogenicity [16]. The Δ*mopex7* mutant is reduced in utilization of short-chain fatty acids and reduced its capacity in conidiation [17]. The* MoPEX5* seems to play more important role than* MoPEX7*, because the Δ*mopex5* mutant exhibits more severe defects than Δ*mopex7*, such as failure to utilize some fatty acids, generation of less turgor, and more sensitivity to H_2_O_2_ pressure. Moreover, distinct defects in developments are also detected in Δ*mopex5*. This phenomenon demonstrated that the PEX5-mediated PTS1 peroxisomal import pathway could be more important than the PEX7-mediated PTS2 peroxisomal import pathway [18]. However, both of the Δ*mopex5 *and Δ*mopex7* mutants lose their pathogenicity.* MoPEX6* is also required for long-chain fatty acids utilization and is essential for pathogenicity. The Δ*mopex6* mutant forms nonmelanized appressoria; as a result, it cannot form the penetration peg and infection hyphae. Additionally, mycelia of Δ*mopex6* are more sensitive to Calcofluor White, suggesting the cell wall of the mutant is defect [28].

### 4.2. Peroxisome Fission and Pathogenesis

Block in peroxisome proliferation can result in failure to increase peroxisome number and impact the peroxisomal metabolism. In* M. oryzae*, deletion of* MoPEX11A* and* PEF1 *can both severely reduce the fatty acids utilization and virulence capacity [20]. However, in contrast to totally loss of virulence in Δ*mopex5*, Δ*mopex6*, Δ*mopex7,* or Δ*mopex19*, the reduction of the virulence in Δ*mopex11A* and Δ*pef1 *is evidently slighter [20, 33]. Other phenotypic defects, such as the melanin layer formation, turgor generation, cell wall integrity, and ROS tolerance, are also slighter in Δ*mopex11A *and Δ*pef1* [20, 33]. These indicate that the* de novo* formation, another way for peroxisome proliferation, can still function or compensate the defects of the peroxisome fission in the Δ*mopex11A *and Δ*pef1* mutants.

### 4.3. Pexophagy and Pathogenesis

In* C. orbiculare*, the Atg26-mediated pexophagy has been proved to be essential for pathogenicity, by rapidly removing redundant peroxisomes after appressoria maturation [13]. The recycling of cellular components required for invasive growth could be the primary cause. However, it seems that the pexophagy process could be dispensable for pathogenicity in* M oryzae*. It has been proved that the* Magnaporthe MoATG26* gene is not involved in pexophagy and is dispensable for virulence [19]. Another gene,* MoSNX41*, obtains the ability to regulate pexophagy in* M. oryzae*, and it plays important roles in pathogenesis. However, its pathogenicity-related function is not relevant to roles in pexophagy, because the yeast ScSnx42 (homolog of MoSnx41) can restore the pexophagy deficiency of Δ*mosnx41*, but cannot recover the defects of pathogenesis [19]. The function of MoSnx41 in conidiation and pathogenesis could be related to Snx41-dependent retrieval trafficking pathway, which may function in gamma-glutamyl cycle and GSH antioxidant production.

### 4.4. Woronin Body and Pathogenesis

Failing to form woronin body would result in multiple phenotypic defects in* M. oryzae*. The Δ*hex1* mutant is normal in mycelial growth, conidiation, and mating processes, but it forms abnormal appressoria, delayed in host penetration and severely blocked in infection hyphae growth [45]. As a result, Δ*hex1* is severely reduced in virulence. Besides, lack of* HEX1* will also result in failure to survive in nitrogen starvation condition, which could explain why the mutant cannot survive in host cells with kinds of cellular damage. The peroxisome *β*-oxidation is not affected in Δ*hex1*, indicating the function of woronin body is distinct from the peroxisomes, although it is derived from the latter [45].

### 4.5. Fatty Acid *β*-Oxidation and Pathogenesis

Peroxisomal *β*-oxidation is one of the chief catabolic processes during fungal infection, which can produce acetyl CoA and energy, as well as glycerol. The glycerol is used to form appressoria turgor, and the acetyl CoA can be used by the glyoxylate cycle to produce nutrient; it also can be used to synthesize melanin and cell wall contents, or other purposes. All of the mentioned products are critical for fungal infection. In* M. oryzae*, the Mfp1 protein involving in peroxisomal *β*-oxidation is proved to play important roles in fatty acid metabolism and pathogenesis [47]. Defects in peroxisome biogenesis will severely block fatty acids *β*-oxidation, and all peroxisome biogenesis and peroxisome fission-related genes are important for fatty acids *β*-oxidation. For example, the* PEX6* disruption mutant is defect in olive oil utilization, cell wall integrity, and appressorial melanization and is lost in penetration capacity [28]. Block in acetyl CoA transportation will lead to similar defect in those mentioned cellular processes. The Δ*pth2* mutant produces less melanin than the wild type and fails to penetrate the host cells and thus is essential for pathogenesis [48]. It cannot grow on lipid substrates.* MoCRC1* is also required for olive oil utilization. The Δ*mocrc1* deletion mutant is severely reduced in penetration and invasive growth. The *β*-oxidation can also occur in mitochondria [49]. However, it seems like that the peroxisomal *β*-oxidation is important for appressoria-mediated penetration, while the mitochondrial *β*-oxidation functions in conidial viability and keeping redox homeostasis during host colonization.

### 4.6. Glyoxylate Cycle and Pathogenesis

The peroxisomal *β*-oxidation produced acetyl CoA should be used by the glyoxylate cycle to provide a mechanism for glucose generation. Glyoxylate cycle enzymes, such as Icl1, are required for full virulence of* M. oryzae*. The expression of* ICL1* is significantly elevated during conidial germination, appressorium formation, and penetration peg formation stages. Correspondingly, the Δ*icl1* mutant is delayed in conidial germination and appressorium formation and retards in cuticle penetration [50].

### 4.7. Redox Homeostasis and Pathogenesis

Accompanied with the degradation of fatty acids, redox homeostasis will be broken, where it is harmful to the fungi and must be rebalanced quickly. Failure in removing redundant oxides may lead to reducing of infection. For example, acetyl-CoA formation resulted in mass NADH, which can be eliminated by reoxidating it to NAD^+^. This reaction is catalyzed by peroxisomal lactate dehydrogenase and needs pyruvate. The pyruvate is generated by the alanine: glyoxylate aminotransferase 1 (Agt1) in* M. oryzae*. The Δ*agt1* mutant fails to penetration via appressoria and totally lost its pathogenicity [51].

## 5. Conclusions and Perspective

As a model plant pathogen, the rice blast fungus* M. oryzae* gains more attention on role of peroxisomes than other pathogens. Considerable progress has been made for us to understand life cycle and functions of the peroxisomes in the filamentous fungi. Knowledge gained from past studies will provide comprehensive understanding in the peroxisomes and may lead to develop novel targets for new drugs against pathogenic fungi. The mechanistic details the peroxisome life cycle and functions are developing rapidly, but how these processes can be well tuned according to the developmental stages and environmental conditions is largely unknown. In the future, efforts should be done to elucidate these regulatory mechanisms.

A large number of* PEX* genes can be found in the genome of* M. oryzae*; the precise roles of these should be further characterized in the future. Another challenge is to reveal the mechanism of* de novo* synthesis and uncover its roles during appressorium formation. The connections between the peroxisomes and other cellular processes or structures should also be addressed. Genome-wide screening of peroxisome-related genes and global analysis of the* PEX* genes can help us to systematically investigate functions and mechanisms of the peroxisomes. The omics approaches can help us to establish the peroxisomal regulatory networks.

## Figures and Tables

**Table 1:** Peroxisome-related genes identified in *M. oryzae*.

Gene	Functions	References
Peroxisome biogenesis		
*MoPEX5*	Receptor of PTS1 peroxisomal matrix proteins	[18]
*MoPEX6*	Peroxisomal matrix protein import	[28]
*MoPEX7*	Receptor of PTS2 peroxisomal matrix proteins	[17, 18]
*MoPEX19*	Peroxisomal membrane proteins import	[16]
Peroxisome fission		
*MoPEX11A/11B/11C*	Peroxisome elongation	[20]
*PEF1*	Peroxisome division	[33]
*MoFis1*	Peroxisome division	[36]
Pexophagy		
*MoSNX41*	Pexophagy and retrieval trafficking	[19]
*MoPEX14*	Pexophagy	[19]
Woronin body		
*HEX1*	Seal the septal pore	[45]
*β*-oxidation		
*MFP1*	Peroxisome *β*-oxidation	[47]
*PTH2*	Acetyl CoA transportation	[48]
*CRAT2*	Acetyl CoA transportation	[48]
*MoCRC1*	Carnitine-acylcarnitine carrier	[49]
Glyoxylate cycle		
*ICL1*	Isocitrate lyase/glyoxylate cycle	[50]
Redox homeostasis		
*AGT1*	Synthesis of the pyruvate	[51]

## References

[1] Smith J. J., Aitchison J. D. (2013). Peroxisomes take shape. *Nature Reviews Molecular Cell Biology*.

[2] van der Klei I. J., Veenhuis M. (2006). Yeast and filamentous fungi as model organisms in microbody research. *Biochimica et Biophysica Acta (BBA)—Molecular Cell Research*.

[3] Platta H. W., Erdmann R. (2007). Peroxisomal dynamics. *Trends in Cell Biology*.

[4] van der Klei I. J., Veenhuis M. (1997). Yeast peroxisomes: function and biogenesis of a versatile cell organelle. *Trends in Microbiology*.

[5] Hu J., Baker A., Bartel B. (2012). Plant peroxisomes: biogenesis and function. *Plant Cell*.

[6] Wanders R. J. A., Waterham H. R. (2006). Biochemistry of mammalian peroxisomes revisited. *Annual Review of Biochemistry*.

[7] Pieuchot L., Jedd G. (2012). Peroxisome assembly and functional diversity in eukaryotic microorganisms. *Annual Review of Microbiology*.

[8] Hynes M. J., Murray S. L., Khew G. S., Davis M. A. (2008). Genetic analysis of the role of peroxisomes in the utilization of acetate and fatty acids in *Aspergillus nidulans*. *Genetics*.

[9] Hynes M. J., Murray S. L., Duncan A., Khew G. S., Davis M. A. (2006). Regulatory genes controlling fatty acid catabolism and peroxisomal functions in the filamentous fungus *Aspergillus nidulans*. *Eukaryotic Cell*.

[10] Managadze D., Würtz C., Wiese S. (2010). Identification of PEX33, a novel component of the peroxisomal docking complex in the filamentous fungus *Neurospora crassa*. *European Journal of Cell Biology*.

[11] Sichting M., Schell-Steven A., Prokisch H., Erdmann R., Rottensteiner H. (2003). Pex7p and Pex20p of *Neurospora crassa* function together in PTS2-dependent protein import into peroxisomes. *Molecular Biology of the Cell*.

[12] Managadze D., Würtz C., Sichting M., Niehaus G., Veenhuis M., Rottensteiner H. (2007). The peroxin PEX14 of *Neurospora crassa* is essential for the biogenesis of both glyoxysomes and woronin bodies. *Traffic*.

[13] Asakura M., Ninomiya S., Sugimoto M. (2009). Atg26-mediated pexophagy is required for host invasion by the plant pathogenic fungus *Colletotrichum orbiculare*. *The Plant Cell*.

[14] Fujihara N., Sakaguchi A., Tanaka S. (2010). Peroxisome biogenesis factor PEX13 is required for appressorium-mediated plant infection by the anthracnose fungus *Colletotrichum orbiculare*. *Molecular Plant-Microbe Interactions*.

[15] Kubo Y., Fujihara N., Harata K., Neumann U., Robin G. P., O'Connell R. (2015). *Colletotrichum orbiculare* FAM1 encodes a novel Woronin body-associated Pex22 peroxin required for appressorium-mediated plant infection. *Mbio*.

[16] Li L., Wang J. Y., Zhang Z. (2014). MoPex19, which is essential for maintenance of peroxisomal structure and woronin bodies, is required for metabolism and development in the rice blast fungus. *PLoS ONE*.

[17] Goh J., Jeon J., Kim K. S., Park J., Park S.-Y., Lee Y.-H. (2011). The PEX7-mediated peroxisomal import system is required for fungal development and pathogenicity in *Magnaporthe oryzae*. *PLoS ONE*.

[18] Wang J. Y., Zhang Z., Wang Y. L. (2013). PTS1 peroxisomal import pathway plays shared and distinct roles to PTS2 pathway in development and pathogenicity of *Magnaporthe oryzae*. *PLoS ONE*.

[19] Deng Y., Qu Z., Naqvi N. I. (2013). The role of Snx41-based pexophagy in *Magnaporthe* development. *PLoS ONE*.

[20] Wang J. Y., Li L., Zhang Z. (2015). One of three Pex11 family members is required for peroxisomal proliferation and full virulence of the rice blast fungus *Magnaporthe oryzae*. *PLoS ONE*.

[21] Piekarska K., Hardy G., Mol E. (2008). The activity of the glyoxylate cycle in peroxisomes of *Candida albicans* depends on a functional *β*-oxidation pathway: evidence for reduced metabolite transport across the peroxisomal membrane. *Microbiology*.

[22] Gabriel F., Accoceberry I., Bessoule J. J. (2014). A Fox2-dependent fatty acid beta-oxidation pathway coexists both in peroxisomes and mitochondria of the ascomycete Yeast *Candida lusitaniae*. *PLoS ONE*.

[23] Gründlinger M., Yasmin S., Lechner B. E. (2013). Fungal siderophore biosynthesis is partially localized in peroxisomes. *Molecular Microbiology*.

[24] Beck J., Ebel F. (2013). Characterization of the major Woronin body protein HexA of the human pathogenic mold *Aspergillus fumigatus*. *International Journal of Medical Microbiology*.

[25] Kretschmer M., Wang J., Kronstad J. W. (2012). Peroxisomal and mitochondrial *β*-oxidation pathways influence the virulence of the pathogenic fungus Cryptococcus neoformans. *Eukaryotic Cell*.

[26] Idnurm A., Giles S. S., Perfect J. R., Heitman J. (2007). Peroxisome function regulates growth on glucose in the basidiomycete fungus *Cryptococcus neoformans*. *Eukaryotic Cell*.

[27] Titorenko V. I., Rachubinski R. A. (2001). The life cycle of the peroxisome. *Nature Reviews Molecular Cell Biology*.

[28] Ramos-Pamplona M., Naqvi N. I. (2006). Host invasion during rice-blast disease requires carnitine-dependent transport of peroxisomal acetyl-CoA. *Molecular Microbiology*.

[29] Hettema E. H., Girzalsky W., Van Den Berg M., Erdmann R., Distel B. (2000). Saccharomyces cerevisiae Pex3p and Pex19p are required for proper localization and stability of peroxisomal membrane proteins. *The EMBO Journal*.

[30] Sacksteder K. A., Jones J. M., South S. T., Li X., Liu Y., Gould S. J. (2000). PEX19 binds multiple peroxisomal membrane proteins, is predominantly cytoplasmic, and is required for peroxisome membrane synthesis. *The Journal of Cell Biology*.

[31] Ghaedi K., Tamura S., Okumoto K., Matsuzono Y., Fujiki Y. (2000). The peroxin Pex3p initiates membrane assembly in peroxisome biogenesis. *Molecular Biology of the Cell*.

[32] Fang Y., Morrell J. C., Jones J. M., Gould S. J. (2004). PEX3 functions as a PEX19 docking factor in the import of class I peroxisomal membrane proteins. *Journal of Cell Biology*.

[33] Chen X.-L., Shen M., Yang J. (2016). Peroxisomal fission is induced during appressorium formation and is required for full virulence of the rice blast fungus. *Molecular Plant Pathology*.

[34] Motley A. M., Ward G. P., Hettema E. H. (2008). Dnm1p-dependent peroxisome fission requires Caf4p, Mdv1p and Fis1p. *Journal of Cell Science*.

[35] Schrader M., Bonekamp N. A., Islinger M. (2012). Fission and proliferation of peroxisomes. *Biochimica et Biophysica Acta (BBA)—Molecular Basis of Disease*.

[36] Khan I. A., Ning G., Liu X., Feng X., Lin F., Lu J. (2015). Mitochondrial fission protein MoFis1 mediates conidiation and is required for full virulence of the rice blast fungus Magnaporthe oryzae. *Microbiological Research*.

[37] Reidick C., Platta H. (2013). Regulation of the selective autophagic degradation of peroxisomes by PtdIns3P and Rab-GTPases. *Yeast*.

[38] Farré J.-C., Manjithaya R., Mathewson R. D., Subramani S. (2008). PpAtg30 tags peroxisomes for turnover by selective autophagy. *Developmental cell*.

[39] Nazarko T. Y., Polupanov A. S., Manjithaya R. R., Subramani S., Sibirny A. A. (2007). The requirement of sterol glucoside for pexophagy in yeast is dependent on the species and nature of peroxisome inducers. *Molecular Biology of the Cell*.

[40] Meijer W. H., van der Klei I. J., Veenhuis M., Kiel J. A. K. W. (2007). ATG genes involved in non-selective autophagy are conserved from yeast to man, but the selective Cvt and pexophagy pathways also require organism-specific genes. *Autophagy*.

[41] Komori M., Rasmussen S. W., Kiel J. A. K. W. (1997). The Hansenula polymorpha PEX14 gene encodes a novel peroxisomal membrane protein essential for peroxisome biogenesis. *The EMBO Journal*.

[42] Liu F. F., Ng S. K., Lu Y. F., Low W., Lai J., Jedd G. (2008). Making two organelles from one: woronin body biogenesis by peroxisomal protein sorting. *The Journal of Cell Biology*.

[43] Trinci A. P. J., Collinge A. J. (1974). Occlusion of the septal pores of damaged hyphae of *Neurospora crassa* by hexagonal crystals. *Protoplasma*.

[44] Markham P., Collinge A. J. (1987). Woronin bodies of filamentous fungi. *FEMS Microbiology Letters*.

[45] Soundararajan S., Jedd G., Li X., Ramos-Pamploña M., Chua N. H., Naqvi N. I. (2004). Woronin body function in *Magnaporthe grisea* is essential for efficient pathogenesis and for survival during nitrogen starvation stress. *Plant Cell*.

[46] Zeng X.-Q., Chen G.-Q., Liu X.-H. (2014). Crosstalk between SNF1 pathway and the peroxisome-mediated lipid metabolism in *Magnaporthe oryzae*. *PLoS ONE*.

[47] Wang Z.-Y., Soanes D. M., Kershaw M. J., Talbot N. J. (2007). Functional analysis of lipid metabolism in *Magnaporthe grisea* reveals a requirement for peroxisomal fatty acid *β*-oxidation during appressorium-mediated plant infection. *Molecular Plant-Microbe Interactions*.

[48] Bhambra G. K., Wang Z.-Y., Soanes D. M., Wakley G. E., Talbot N. J. (2006). Peroxisomal carnitine acetyl transferase is required for elaboration of penetration hyphae during plant infection by *Magnaporthe grisea*. *Molecular Microbiology*.

[49] Yang J., Kong L., Chen X. (2012). A carnitine-acylcarnitine carrier protein, MoCrc1, is essential for pathogenicity in *Magnaporthe oryzae*. *Current Genetics*.

[50] Wang Z.-Y., Thornton C. R., Kershaw M. J., Debao L., Talbot N. J. (2003). The glyoxylate cycle is required for temporal regulation of virulence by the plant pathogenic fungus *Magnaporthe grisea*. *Molecular Microbiology*.

[51] Bhadauria V., Banniza S., Vandenberg A., Selvaraj G., Wei Y. (2012). Peroxisomal alanine: glyoxylate aminotransferase AGT1 is indispensable for appressorium function of the rice blast pathogen, magnaporthe oryzae. *PLoS ONE*.

[52] Kunze M., Pracharoenwattana I., Smith S. M., Hartig A. (2006). A central role for the peroxisomal membrane in glyoxylate cycle function. *Biochimica et Biophysica Acta—Molecular Cell Research*.

[53] Opaliński L., Bartoszewska M., Fekken S. (2012). *De novo* peroxisome biogenesis in *Penicillium chrysogenum* is not dependent on the Pex11 family members or Pex16. *PLoS ONE*.

[54] Langfelder K., Streibel M., Jahn B., Haase G., Brakhage A. A. (2003). Biosynthesis of fungal melanins and their importance for human pathogenic fungi. *Fungal Genetics and Biology*.

[55] Poirier Y., Antonenkov V. D., Glumoff T., Hiltunen J. K. (2006). Peroxisomal *β*-oxidation—a metabolic pathway with multiple functions. *Biochimica et Biophysica Acta—Molecular Cell Research*.

[56] Patkar R. N., Ramos-Pamplona M., Gupta A. P., Fan Y., Naqvi N. I. (2012). Mitochondrial *β*-oxidation regulates organellar integrity and is necessary for conidial germination and invasive growth in *Magnaporthe oryzae*. *Molecular Microbiology*.

[57] Swiegers J. H., Dippenaar N., Pretorius I. S., Bauer F. F. (2001). Carnitine-dependent metabolic activities in *Saccharomyces cerevisiae*: three carnitine acetyltransferases are essential in a carnitine-dependent strain. *Yeast*.

[58] Titorenko V. I., Terlecky S. R. (2011). Peroxisome metabolism and cellular aging. *Traffic*.

[59] Skamnioti P., Henderson C., Zhang Z., Robinson Z., Gurr S. J. (2007). A novel role for catalase B in the maintenance of fungal cell-wall integrity during host invasion in the rice blast fungus *Magnaporthe grisea*. *Molecular Plant-Microbe Interactions*.

[60] Tanabe S., Ishii-Minami N., Saitoh K.-I. (2011). The role of catalase-peroxidase secreted by *Magnaporthe oryzae* during early infection of rice cells. *Molecular Plant-Microbe Interactions*.

[61] Schrader M., Fahimi H. D. (2006). Peroxisomes and oxidative stress. *Biochimica et Biophysica Acta—Molecular Cell Research*.

[62] Kim K.-H., Willger S. D., Park S.-W. (2009). TmpL, a transmembrane protein required for intracellular redox homeostasis and virulence in a plant and an animal fungal pathogen. *PLoS Pathogens*.

[63] Hamilton A. J., Gomez B. L. (2002). Melanins in fungal pathogens. *Journal of Medical Microbiology*.

[64] Bowman S. M., Free S. J. (2006). The structure and synthesis of the fungal cell wall. *BioEssays*.

